# Supplementary material for: COVID-19 vaccination uptake in 441 socially and ethnically diverse pregnant women
Source: PLoS One. 2022 Aug 17;17(8):e0271834. doi: 10.1371/journal.pone.0271834 (PMC9385003; doi:10.1371/journal.pone.0271834)
Supplement: S1 Fig — (PDF) [file pone.0271834.s001.pdf]

Figure S1: Anonymous Questionnaire among pregnant women in Frimley NHS Trust

NHS
Frimley Health
NHS Foundation Trust

COVID-19 Vaccine in Pregnancy- Patient Survey

Frimley Health is conducting a short survey to understand pregnant women's attitudes towards, and opinion of, the COVID-19 vaccine. We would be really grateful if you would take a couple of minutes to complete this brief survey. If you would prefer to complete this survey on your phone or other mobile device please scan the QR code above. This survey is completely anonymous. Thank you in advance for your time!

The Obstetrics Department Team

Today's Date: / /

1) Which is your local hospital?

- ☐ Frimley Park
- ☐ Wexham Park

2) Have you previously tested positive for COVID-19?

- ☐ Yes → Go to **Q3**
- ☐ No → Go to **Q5**
- ☐ Don't know / can't remember → **Q5**

3) If you have previously tested positive for COVID-19 when was it?

Month: ___________________________

Year: ___________________________

4) Were you pregnant at the time?

- ☐ Yes
- ☐ No

5) Have you received a COVID-19 vaccine?

- ☐ Yes → Go to **Q6**
- ☐ No – I chose not to → Go to **Q8**
- ☐ No – I was advised not to→ Go to **Q10**
- ☐ Don't know / can't remember → Go to **Q10**

6) Which vaccine did you receive?

- ☐ AstraZeneca
- ☐ Moderna
- ☐ Pfizer
- ☐ Don't know / can't remember

7) How many doses have you received?

- ☐ 1 → Go to **Q10**
- ☐ 2 → Go to **Q10**
- ☐ Don't know / can't remember → Go to **Q10**

Survey continues overleaf →

8) If you have not been vaccinated, what are some of the reasons you may not want to receive a COVID vaccination? (Please check all that apply)

- ☐ Concerns about fertility
- ☐ Concerns about the vaccine's effects on baby/future pregnancies
- ☐ Concerns about breastfeeding after receiving vaccine
- ☐ My midwife / obstetrics doctor / GP did not offer the vaccine to me
- ☐ My midwife / obstetrics doctor / GP told me I was not in a COVID-19 high risk group
- ☐ Concerns about vaccine safety for myself
- ☐ Previously had COVID-19
- ☐ Not enough information known about vaccine
- ☐ Other → Go to **Q9**

9) What other concerns do you have about the COVID-19 vaccine?

10) Where do you get information about the vaccine? (Please check all that apply)

- ☐ My local GP / community midwife
- ☐ Friends and family
- ☐ Mainstream news organisations – e.g. BBC, ITV, newspapers
- ☐ Social Media – e.g. Facebook, Instagram, YouTube, TikTok etc.
- ☐ Specific health-related websites – e.g. www.nhs.uk, www.fhft.org.uk

11) If you get information from other sources please let us know:

12) Which single source do you trust the most to give information about the vaccine? (Please select a single option)

- ☐ My local GP / community midwife
- ☐ Friends and family
- ☐ Mainstream news organisations – e.g. BBC, ITV, newspapers
- ☐ Social Media – e.g. Facebook, Instagram, YouTube, TikTok etc.
- ☐ Specific health-related websites – e.g. www.nhs.uk, www.fhft.org.uk
- ☐ Other → **Q13**

13) If you prefer to trust another source of information please let us know:

NHS
Frimley Health
NHS Foundation Trust

14) **Do you have any underlying medical issues? (Please check all that apply)**

- ☐ None
- ☐ Diabetes
- ☐ High Blood Pressure
- ☐ Hypertension
- ☐ Asthma
- ☐ High cholesterol
- ☐ Kidney Disease
- ☐ Heart Condition → Go to **Q15**
- ☐ Other → Go to **Q16**

15) **Please specify your heart condition (If none please leave blank):**

16) **Please specify any other underlying health condition(s) (If none please leave blank):**

17) **How many children have you given birth to?**

18) **How many weeks pregnant are you?**

19) **What age are you? Please leave blank if you do not wish to disclose**

20) **What ethnic group are you?**

- ☐ White British
- ☐ White other
- ☐ Mixed/multiple ethnic groups
- ☐ Asian or Asian British
- ☐ Black/African/Caribbean/Black British
- ☐ Other ethnic group
- ☐ I do not wish to disclose

21) **Is English your main language?**

- ☐ Yes → **End of survey – Thank you**
- ☐ No → Go to **Q22**
- ☐ I do not wish to disclose → **End of survey – Thank you**

22) **What is your main language?**

Thank you very much for your time – we really appreciate it.
