# Supplementary material for: COVID-19 vaccination uptake in 441 socially and ethnically diverse pregnant women
Source: PLoS One. 2022 Aug 17;17(8):e0271834. doi: 10.1371/journal.pone.0271834 (PMC9385003; doi:10.1371/journal.pone.0271834)
Supplement: Attachment — Submitted filename: Response to editors and reviewers.docx [file pone.0271834.s002.docx]

**RESPONSE TO THE EDITORS AND REVIEWERS –PONE-D-22-11364**

**We thank the reviewers and editors for their thoughtful comments which we think have improved the paper. Please find below our response to the points raised.**

| **COMMENT** | **RESPONSE** | **EDITS IN MANUSCRIPT** |
| --- | --- | --- |
| **Editors’ comments to authors** |  |  |
| Specifically, these areas are in need of revision, clarification, and improvement:  Design of the study should be revised  Introduction section should be expanded  Results should be improved:  The numbers of women attending the hospital and clinics during the period of the study.  The percentage of women who did not respond to invitation to participate in the study  The percentage of women who did not give their consent to participate  Discussion section should be more robust:    The rate of other pregnancy vaccines uptake in the study hospitals for comparison. Comparisons with recent similar works across continents and comparison with national vaccine uptake | As suggested, we have clarified the design, expanded the introduction and discussion sections and have provided additional information for the estimated response rates and pertussis vaccine provided during pregnancy. | Throughout the manuscript |

| 1. Please ensure that your manuscript meets PLOS ONE's style requirements, including those for file naming.   2. Please provide additional details regarding participant consent. In the ethics statement in the Methods and online submission information, please ensure that you have specified (1) whether consent was informed and (2) what type you obtained (for instance, written or verbal, and if verbal, how it was documented and witnessed). If your study included minors, state whether you obtained consent from parents or guardians. If the need for consent was waived by the ethics committee, please include this information.  If you are reporting a retrospective study of medical records or archived samples, please ensure that you have discussed whether all data were fully anonymized before you accessed them and/or whether the IRB or ethics committee waived the requirement for informed consent. If patients provided informed written consent to have data from their medical records used in research, please include this information.  3. Thank you for stating the following in the Acknowledgments Section of your manuscript:  " MSR is funded with a NIHR In-practice Fellowship (NIHR 302007). "  We note that you have provided funding information that is not currently declared in your Funding Statement. However, funding information should not appear in the Acknowledgments section or other areas of your manuscript. We will only publish funding information present in the Funding Statement section of the online submission form.  Please remove any funding-related text from the manuscript and let us know how you would like to update your Funding Statement. Currently, your Funding Statement reads as follows:  " The authors received no specific funding for this work"  Please include your amended statements within your cover letter; we will change the online submission form on your behalf.  4. We note that you have stated that you will provide repository information for your data at acceptance. Should your manuscript be accepted for publication, we will hold it until you provide the relevant accession numbers or DOIs necessary to access your data. If you wish to make changes to your Data Availability statement, please describe these changes in your cover letter and we will update your Data Availability statement to reflect the information you provide.  5. Please include your full ethics statement in the ‘Methods’ section of your manuscript file. In your statement, please include the full name of the IRB or ethics committee who approved or waived your study, as well as whether or not you obtained informed written or verbal consent. If consent was waived for your study, please include this information in your statement as well.  6. Please include captions for your Supporting Information files at the end of your manuscript, and update any in-text citations to match accordingly.  Please see our Supporting Information guidelines for more information: <http://journals.plos.org/plosone/s/supporting-information>.  7. Please review your reference list to ensure that it is complete and correct. If you have cited papers that have been retracted, please include the rationale for doing so in the manuscript text, or remove these references and replace them with relevant current references. Any changes to the reference list should be mentioned in the rebuttal letter that accompanies your revised manuscript. If you need to cite a retracted article, indicate the article’s retracted status in the References list and also include a citation and full reference for the retraction notice. | Manuscript revised to ensure it meets the PLOS ONE guidelines.  Consent information provided in the Methods section as suggested  This information is removed from the acknowledgement section and will be included in the cover letter. Please note that MSR’s NIHR In-Practice fellowship funding is a research training fellowship and is not specifically for this work.  All data will be deposited to St George’s University’s repository (***Figshare)*** and a link will be provided to the journal if the manuscript is accepted for publication.  Ethics and informed consent information is included in the Methods section as suggested.  Supplementary information (S1 Figure) provided in a separate document with title “S1_fig.pdf”  Done as suggested | Pages 4-5  Page 4  Supplementary PDF  Pages 13-15 |
| --- | --- | --- |
| **Reviewers’ comments to the authors** | **Response** | **Edits in manuscript** |
| Although there have been a number of national surveys investigating uptake of COVID-19 among pregnant women, it is important to understand the local situation with respect to vaccines uptake locally. This is a clearly written paper describing a study set in two English hospitals.  I would suggest expanding the introduction section to include some detail about the original recommendation re COVID-19 vaccine in pregnancy ie that it was not recommended routinely <https://www.gov.uk/government/publications/priority-groups-for-coronavirus-covid-19-vaccination-advice-from-the-jcvi-30-december-2020/joint-committee-on-vaccination-and-immunisation-advice-on-priority-groups-for-covid-19-vaccination-30-december-2020>. This is important to set the context for the reader who may not be familiar with the change in advice (eg an International reader) and also because some women refer to this as their reason for not accepting the vaccine.  Due to the methods used it is understandable that no response rate could be calculated. However, some estimation could be made as the numbers of women attending during the period of the study must be known, equally information on ethnicity generally in this population and of chronic health conditions so that some comment on representativeness could be made.  My main comments are with the description of the type of study. This is ostensibly a survey and although open ended responses were 'analysed thematically' and quotes included, I would suggest this does not make this a mixed methods study, nor the open ended data 'qualitative' in which you would expect in depth interviews to have been conducted in addition to the survey.  In the section on information sources, it is reported that the single most trusted sources of information were the patient's local GP or midwife. This is a common finding in studies of vaccination uptake. On this basis, the authors then go on to recommend that trusted sources of information should be used to address concerns about vaccine safety and long-term vaccine safety. While this is not unreasonable, I would suggest that a trusted sources does not necessarily equal an accurate source. Indeed, it has been shown that midwives themselves often do not accept COVID-19 vaccine in fact in the SIREN study, they were the health staff group with the highest non acceptance (https://www.sciencedirect.com/science/article/pii/S014067362100790X). another study (not peer reviewed) found important differences in uptake among midwives with a minority ethnic background (https://assets.researchsquare.com/files/rs-646142/v1/f151867e-9f92-48eb-b554-bea59db31657.pdf?c=1631888999) and so I would be inclined to explore further with midwives, their views about COVID- 19 vaccine. Training of staff is not mentioned in the recommendations, which should be a priority. It would be useful to look at the data in more detail to explore this issue perhaps a cross tab of 'most trusted source' x vaccine uptake - though probably not possible as GP and community midwife are one category.  Are data available on uptake of other pregnancy vaccines in the study hospitals for comparison? Skirrow et al (ref 3) found an association between non acceptance of pertussis vaccine and COVID vaccine among pregnant women. In addition, a comparison with national uptake would be useful (53.7% on 24th March 2022)  A few minor details: Page 5 - last but last line 'by an experienced statistician' is not needed Page 6 - missing full stop after syndrome Page 9 - second para - typo heath  Reviewer #2: The study is relevant as it is important to explore the views of special groups of people (in this case pregnant women) towards COVID-19 vaccination. Find some comments below:  Abstract Kindly rephrase the method by removing "we". It can be stated as "Consented pregnant women completed the electronic survey using a QR code or on paper. Data were summarised descriptively, and text responses were thematically analysed."  Discussion Discussion section should be more robust. Focusing on the objectives, vaccination uptake by pregnant women facilitators and barriers with factors responsible for these can be comprehensively discussed. Significance of ethnic diversity can be discussed. Discussion of present find with literature summarised under introduction can also be explained. | We thank the reviewer for the feedback and very helpful suggestions. We have incorporated all the suggestions in the manuscript.  Added as suggested.  We have provided estimates for the response rate where possible as well as discussion of the number of high-risk patients in pregnant women across the two sites.  We have changed the title as suggested  We have included the papers as suggested and discussed the reported studies about higher vaccine hesitancy amongst midwives in particular ethnic minority midwives.  Training of staff added to the list of recommendations as suggested. Unfortunately, the survey did not collect disaggregated data on trusted sources of information (i.e midwives and GPs).  We have added the latest data from UK Health Security Agency for comparison as well as data for pertussis vaccination from both sites in 2021.  We have corrected the errors and made the changes as suggested.  We thank the reviewer for the feedback and very helpful suggestions. We have incorporated all the suggestions in the manuscript.  This is amended as suggested.  We have revised and expanded the discussion as suggested. | Page 3, paragraph 1  Pages 5 and 11  Title page  Pages 10-11  Pages 11 and 19 (Table 3)  Pages 3 and 10  Page 2  Pages 9-12 |
